# APOE4 promotes nigral tau hyperphosphorylation through cholesterol in atherosclerosis

**DOI:** 10.1038/s41420-025-02778-1

**Published:** 2025-10-21

**Authors:** Shanshan Hu, Xiaojia Peng, Bing Xia, Cihang Gu, Baofei Sun, Min Chang, Jiuyang Ding, Longying Peng

**Affiliations:** 1 Good Clinical Practice Center, Affiliated Hospital of Zunyi Medical University, Zunyi, 563000 China; 2 Department of Neurology, The Third Affiliated Hospital, Southern Medical University, Guangzhou, 510630 China; 3 School of Forensic Medicine, Guizhou Medical University, Guiyang, 550025 China; 4 Guangzhou Key Laboratory of Forensic Multi-Omics for Precision Identification, School of Forensic Medicine, Southern Medical University, Guangzhou, 510515 China; 5 Key Laboratory of Human Brain bank for Functions and Diseases of Department of Education of Guizhou Province, Guizhou Medical University, Guiyang, 550025 China; 6 Department of Pediatrics, Affiliated Hospital of Zunyi Medical University, Zunyi, 563000 China; 7 Department of Pediatrics, Guizhou Children’s Hospital, Zunyi, 563000 China

**Keywords:** Parkinson's disease, Atherosclerosis

## Abstract

Nigral tau hyperphosphorylation has been implicated as an initiation of nigrostriatal dopaminergic neurodegeneration. Apolipoprotein epsilon 4 allele (APOE4) is a common risk factor of Parkinson’s disease (PD) and atherosclerosis (AS). Whether APOE4 carriers exhibited higher levels of nigral phosphorylated tau (p-tau) and the correlation between AS- and PD-related tauopathy remain elusive. Here, the tau pathology was observed in APOE4 carried and non-APOE4 carried AS patients postmortem brain substantia nigra pars compacta (SNpc). APOE3/3 and APOE4/4 knock-in mice treated with high fat diet (APOE3-HFD and APOE4-HFD, respectively) were used to simulate AS model. The tau-related neuropathology and behavioral performances were analyzed. Postmortem brain analysis showed that APOE4-carried AS patients exhibited elevated nigral p-tau level relative to non-APOE4 carriers. APOE4 mice fed with HFD exhibited higher p-tau, cholesterol accumulation, and larger AS plaque area in contrast to APOE3-HFD. Cholesterol triggered GSK3β activation, leading to tau phosphorylation in primary cultured neurons. Aiding cholesterol transport alleviated nigral cholesterol accumulation and tau pathology, thereby mitigating the tau-mediated nigrostriatal degeneration. This alleviated degeneration might also contribute to motor function recovery. These findings showed a link between nigral dopaminergic tau-related pathology and AS phenotype, and targeting cholesterol might alleviate both PD-like tauopathy and AS.

## Introduction

Unstable atherosclerosis (AS) plaque rupture, which belongs to the thromboembolic events, remains the leading cause of cardiovascular mortality especially in the coronary artery and cerebral circle of Willis [1]. Parkinson’s disease (PD) is one of the most common neurodegenerative disease and affects ~3% of the elderly [2]. Recent studies showed a vital link between AS and PD morbidity [3, 4]. AS and PD shared similar pathophysiological processes such as inflammation, homeostatic imbalance of cholesterol, and oxidative stress [5, 6]. However, characteristics of PD related pathology in AS postmortem human brains remain elusive.

The PD pathology was characterized by Lewy bodies and Lewy neurites which contained phosphorylated ɑ-synuclein (ɑ-syn) aggregates, dopaminergic neuron loss in substantial nigra pars compacta (SNpc) [7]. A recent study emphasized a role of microtubule associated protein tau (MAP tau, tau) pathology in PD and Parkinsonism, suggesting dopaminergic neurodegeneration can be phosphorylated tau (p-tau) mediated beside of ɑ-syn [8, 9]. Tau and α-synuclein can also assemble into condensates, aggregates and droplets via liquid-liquid phase separation, leading to amyloid fibril formation in PD and AD [10, 11]. Higher CSF p-tau levels were correlated with morbidity of motor complications [12]. Although PD was not a typical tauopathy, half of PD patient brains showed tau pathology [13, 14]. Whether tau pathology appeared in postmortem AS patient brains had not been addressed before.

The *E4* allele variant of the *APOE* gene (*APOE4*) was considered as a common genetic risk factor for AS and PD [3, 15]. The *APOE4* carriers had a plasma protein phenotype linked to AS [16]. And the *APOE* genotype was associated with tau burden in postmortem human brain analysis [17]. Whether *APOE4* carried AS patient brains had more tau inclusions relative to non-*APOE4* carriers had not been illustrated earlier. As APOE4 was related to cholesterol accumulation [18–21], and tau hyperphosphorylation was kinase dependent [22, 23], we sought to uncover the correlations between cholesterol and tau kinase protein levels.

Neurofibrillary tangels (NFTs), consisting of insoluble p-tau aggregates, were characterized for neuropathological features of PD and AD [8]. We used AT8 antibody readout, which recognized Ser202 and Thr205 site of p-tau, to visualize NTFs. However, early stages of p-tau assemblies were consisted of other sites of p-tau, such as Ser396, Thr217 etc. These sites of p-tau showed superiorities for predication before neuropathology onset [24]. The early stage of p-tau and NFTs had important meanings to reveal the pathology of neurodegenerative disease. Here, we firstly tested the tau pathology, tau phosphorylation at multiple sites, in AS postmortem brain SNpc of *APOE4* carriers and non-*APOE4* carriers. Next, we used *APOE4/4* homozygote (hereafter APOE4 mice) fed with high fat diet (HFD) to simulate AS model, and the *APOE3/3* homozygote mice (APOE3 mice) was used for negative control. By analyzing the p-tau levels and motor performances in AS mice, we sought to uncovered the mechanistic links between APOE4 and nigral tau pathology. Our study showed that tau hyperphosphorylation was more evident in APOE4 carried postmortem nigral regions relative to non-APOE4 carriers. Neuronal cholesterol accumulation triggered tau hyperphosphorylation through activating tau kinase GSK3β, leading to nigrostriatal degeneration and motor performance impairments in APOE4-HFD mice. Facilitating cholesterol transport alleviated PD related pathology and motor impairment in AS mice model. Our study revealed a potential link between AS and PD pathology and provided evidence for PD.

## Results

### More potent p-tau accumulation and neuropathology in the SNpc of APOE4-carried AS patients

**Fig. 1:**
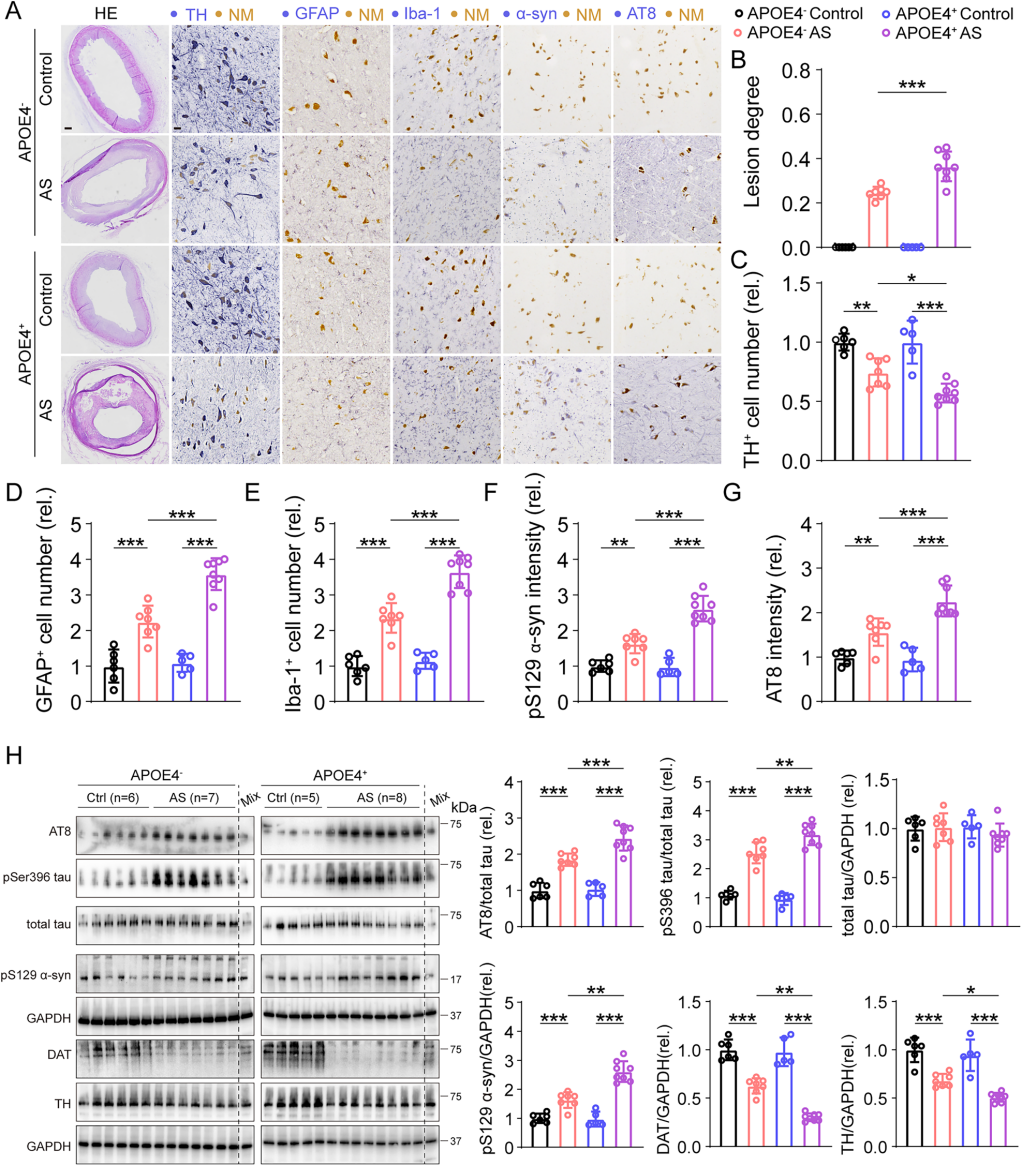
Dopaminergic neurodegeneration and higher levels of p-tau in APOE4 carried AS patients postmortem SNpc. **A** HE staining of coronary arteries (left panel), and TH, GFAP, Iba-1, AT8 and pSer396 tau IHC staining (right five panels) in SNpc in postmortem samples. NM, neuroelanin, note that the yellow-brown colored NM exists only in the dopaminergic neurons in SNpc. Bar in HE, 500 μm; Bar in all IHC staining panels, 10 μm. **B** Lesion degree of coronary arteries in AS and healthy controls. *n* = 5–8, two-way ANOVA followed by Bonferroni’s post hoc tests. Phenotype: *F*(1, 22) = 489.1, *p* < 0.001, Genotype: *F*(1, 22) = 22.43, *p* < 0.001, Phenotype × Genotype: *F*(1, 22) = 22.43, *p* < 0.001. **C** Number of TH positive cells in SNpc of postmortem brain tissues. *n* = 5–8, two-way ANOVA followed by Bonferroni’s *post ho*c tests. Phenotype: *F*(1, 22) = 92.53, *p* < 0.001, Genotype: *F*(1, 22) = 5.978, *p* = 0.021, Phenotype × Genotype: *F*(1, 22) = 5.978, *p* = 0.021. **D** Number of GFAP positive cells in SNpc of postmortem brain tissues. *n* = 5–8, two-way ANOVA followed by Bonferroni’s post hoc tests. Phenotype: *F*(1, 22) = 200.4, *p* < 0.001, Genotype: *F*(1, 22) = 27.13, *p* < 0.001, Phenotype × Genotype: *F*(1, 22) = 22.74, *p* < 0.001. **E** Number of Iba-1 positive cells in SNpc of postmortem brain tissues. *n* = 5–8, two-way ANOVA followed by Bonferroni’s post hoc tests. Phenotype: *F*(1, 22) = 266.2, *p* < 0.001, Genotype: *F*(1, 22) = 37.31, *p* < 0.001, Phenotype × Genotype: *F*(1, 22) = 23.58, *p* < 0.001. **F** Relative intensity of pSer129 α-syn in SNpc. *n* = 5–8, two-way ANOVA followed by Bonferroni’s post hoc tests. Phenotype: *F*(1, 22) = 166.9, *p* < 0.001, Genotype: *F*(1, 22) = 29.82, *p* < 0.001, Phenotype × Genotype: *F*(1, 22) = 32.87, *p* < 0.001. **G** Relative intensity of AT8 in SNpc. *n* = 5–8, two-way ANOVA followed by Bonferroni’s post hoc tests. Phenotype: *F*(1, 22) = 110.0, *p* < 0.001, Genotype: *F*(1, 22) = 12.83, *p* = 0.001, Phenotype × Genotype: *F*(1, 22) = 17.50, *p* < 0.001. **H** Representative blots and quantification of phosphorylated tau at multiple sites and pSer129 α-syn in SNpc of postmortem brain tissues. two-way ANOVA followed by Bonferroni’s post hoc tests. AT8, Phenotype: *F*(1, 22) = 207.1, *p* < 0.001, Genotype: *F*(1, 22) = 16.79, *p* < 0.001, Phenotype × Genotype: *F*(1, 22) = 12.69, *p* = 0.001, pS396 tau, Phenotype: *F*(1, 22) = 356.8, *p* < 0.001, Genotype: *F*(1, 22) = 5.198, *p* = 0.03, Phenotype × Genotype: *F*(1, 22) = 13.19, *p* = 0.001, total tau, Phenotype: *F*(1, 22) = 0.769, *p* = 0.387, Genotype: *F*(1, 22) = 0.591, *p* = 0.448, Phenotype × Genotype: *F*(1, 22) = 1.627, *p* = 0.212, pS129 α-synuclein, Phenotype: *F*(1, 22) = 132.5, *p* < 0.001, Genotype: *F*(1, 22) = 11.51, *p* = 0.002, Phenotype × Genotype: *F*(1, 22) = 9.898, *p* = 0.003, DAT, Phenotype: *F*(1, 22) = 319.0, *p* < 0.001, Genotype: *F*(1, 22) = 34.34, *p* < 0.001, Phenotype × Genotype: *F*(1, 22) = 26.83, *p* < 0.001, TH, Phenotype: *F*(1, 22) = 138.2, *p* < 0.001, Genotype: *F*(1, 22) = 13.04, *p* = 0.001, Phenotype × Genotype: *F*(1, 22) = 3.441, *p* = 0.074. *p < 0.05, **p < 0.01, and ***p < 0.001.

APOE4 had been shown a risk factor for AS and PD. We first collected postmortem brains and coronary arteries of AS patients, either as APOE4 carriers and non-APOE4 carriers (APOE4^+^ and APOE4^-^, respectively). The original lumen and plaque-induced narrowed lumen were indicated using black and red dot line, respectively in HE staining coronary artery sections. We noted that the stenosis degree of APOE4^+^ AS patients coronary arteries were higher than that in APOE4^-^ AS patients (Fig. 1A, B).

To access whether APOE4 genotype is essential for maintaining nigral integrity of AS patients, we conducted TH IHC staining in SNpc, and found that DA neuron loss was more server in APOE4^+^ AS patients than that in APOE4^-^ AS patients (Fig. 1A, C). The number of GFAP and Iba-1 positive cell were increased in APOE4^+^ AS SNpc than that in APOE4^-^ (Fig. 1A, D, E). Moreover, the immunoreactivity of pSer129 α-syn, which accounted for Lewy body formation, appeared in AS patients’ nigral region, but not in non-AS brains. And higher intensity of pSer129 α-syn were observed in APOE^+^ versus APOE4^-^ patients (Fig. 1A, F).

It has been shown that APOE4 accounts partly for tau hyperphosphorylation [25], we stained SNpc area using AT8 antibody, a common used antibody for markering neurofibrillary tangles. AS, but not non-AS patients’s nigral regions, showed AT8 positive signals (Fig. 1A, G). Increased AT8 immunoreactivity were seen in APOE4^+^ AS patients’ brains compared to APOE4^-^. The results of blots showed a similar pattern to the IHC images. We found tau hyperphosphorylation at pSer396 and AT8 (pSer202 & pThr205) were more potent in APOE4^+^ AS patients’ brains compared to APOE4^-^. And levels of dopaminergic markers including DAT and TH were decreased in APOE4^+^ AS patients’ brains versus APOE4^-^ (Fig. 1H).

### APOE4 exacerbated tau pathology in mice fed with HFD

**Fig. 2:**
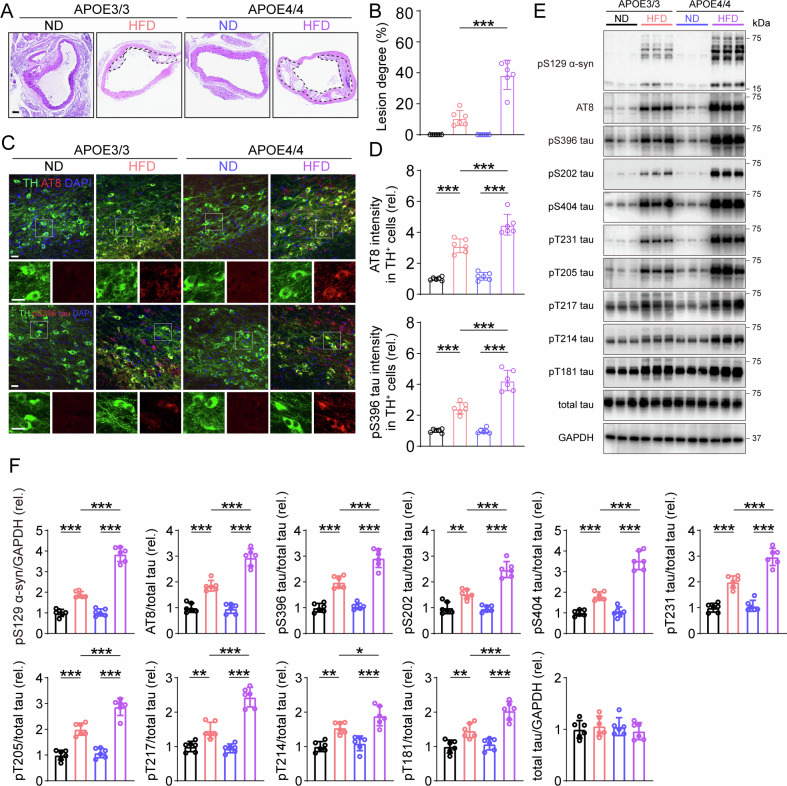
More potent tau hyperphosphorylation in APOE4/4 carried AS mice model. **A** Representative HE staining of coronary arteries. Bar, 20 μm. **B** Lesion degree of coronary arteries in AS mice. Black dashed line showed that area of plaque. *n* = 6, two-way ANOVA followed by Bonferroni’s post hoc tests. Bar, 100 μm. Treatment: *F*(1, 20) = 129.4, *p* < 0.001, Genotype: *F*(1, 20) = 40.9, *p* < 0.001, Phenotype × Genotype: *F*(1, 20) = 40.9, *p* < 0.001. **C** Representative IF staining of TH, AT8 and pSer396 tau in mice SNpc. Bar, **D** Relative intensity of AT8 and pSer396 tau in TH positive cells. *n* = 6, two-way ANOVA followed by Bonferroni’s post hoc tests. AT8, Treatment: F(1, 20) = 247.7, *p* < 0.001, Genotype: F(1, 20) = 19.17, *p* < 0.001, Phenotype × Genotype: F(1, 20) = 12.16, *p* < 0.001. pSer396, Treatment: F(1, 20) = 217.2, *p* < 0.001, Genotype: F(1, 20) = 31.13, *p* < 0.001, Phenotype × Genotype: F(1, 20) = 32.19, *p* < 0.001. **E**, **F** Representative blots and quantification of phosphorylated tau at multiple sites and pSer129 α-syn in SNpc of postmortem brain tissues. two-way ANOVA followed by Bonferroni’s post hoc tests. pS129 α-syn, Treatment: *F*(1, 20) = 402.7, *p* < 0.001, Genotype: *F*(1, 20) = 114.1, *p* < 0.001, Phenotype × Genotype: *F*(1, 20) = 109.8, *p* < 0.001, AT8, Treatment: *F*(1, 20) = 247.7, *p* < 0.001, Genotype: *F*(1, 20) = 19.17, *p* < 0.001, Treatment × Genotype: *F*(1, 20) = 12.16, *p* = 0.002, pS396 tau, Treatment: *F*(1, 20) = 216.6, *p* < 0.001, Genotype: *F*(1, 20) = 25.49, *p* < 0.001, Treatment × Genotype: *F*(1, 20) = 20.25, *p* < 0.001, pS202 tau, Treatment: *F*(1, 20) = 126.5, *p* < 0.001, Genotype: *F*(1, 20) = 25.43, *p* < 0.001, Treatment × Genotype: *F*(1, 20) = 29.25, *p* < 0.001, pS404 tau, Treatment: *F*(1, 20) = 204.7, *p* < 0.001, Genotype: *F*(1, 20) = 58.46, *p* < 0.001, Treatment × Genotype: *F*(1, 20) = 52.68, *p* < 0.001, pT231 tau, Treatment: *F*(1, 20) = 208.1, *p* < 0.001, Genotype: *F*(1, 20) = 26.87, *p* < 0.001, Treatment × Genotype: *F*(1, 20) = 19.85, *p* < 0.001, pT205, Treatment: *F*(1, 20) = 195.7, *p* < 0.001, Genotype: *F*(1, 20) = 22.79, *p* < 0.001, Treatment × Genotype: *F*(1, 20) = 15.40, *p* < 0.001, Pt217, Treatment: *F*(1, 20) = 131.4, *p* < 0.001, Genotype: *F*(1, 20) = 28.59, *p* < 0.001, Phenotype × Genotype: *F*(1, 20) = 35.64, *p* < 0.001, pT214, Treatment: *F*(1, 20) = 61.87, *p* < 0.001, Genotype: *F*(1, 20) = 6.472, *p* = 0.019, Treatment × Genotype: *F*(1, 20) = 2.122, *p* = 0.160, pT181, Treatment: *F*(1, 20) = 68.50, *p* < 0.001, Genotype: *F*(1, 20) = 14.23, *p* = 0.001, Treatment × Genotype: *F*(1, 20) = 8.559, *p* = 0.008, total tau, Treatment: *F*(1, 20) = 0.022, *p* = 0.882, Genotype: *F*(1, 20) = 0.097, *p* = 0.758, Treatment × Genotype: *F*(1, 20) = 1.104, *p* = 0.306. *p < 0.05, **p < 0.01, and ***p < 0.001.

To simulate AS pathology of patients in mice, we treated APOE3 and APOE4 mice with HFD. HE staining showed the lesion degree was higher in APOE4-HFD mice versus APOE3-HFD (Fig. 2A, B). Next, we examined p-tau levels in DA neurons by double immunostaining. Increased AT8 and pSer396 tau levels were observed in APOE4-HFD mice versus APOE3-HFD (Fig. 2C, D). Correlated with the IF staining results, increased levels of p-tau at multiple sites were observed in APOE4-HFD mice compared with APOE3-HFD. No major differences were found in levels of p-tau in APOE3-HFD and APOE4-HFD mice (Fig. 2E, F).

### APOE4 mice treated with HFD caused defects in the nigrostriatal dopaminergic pathway and motor impairments

**Fig. 3:**
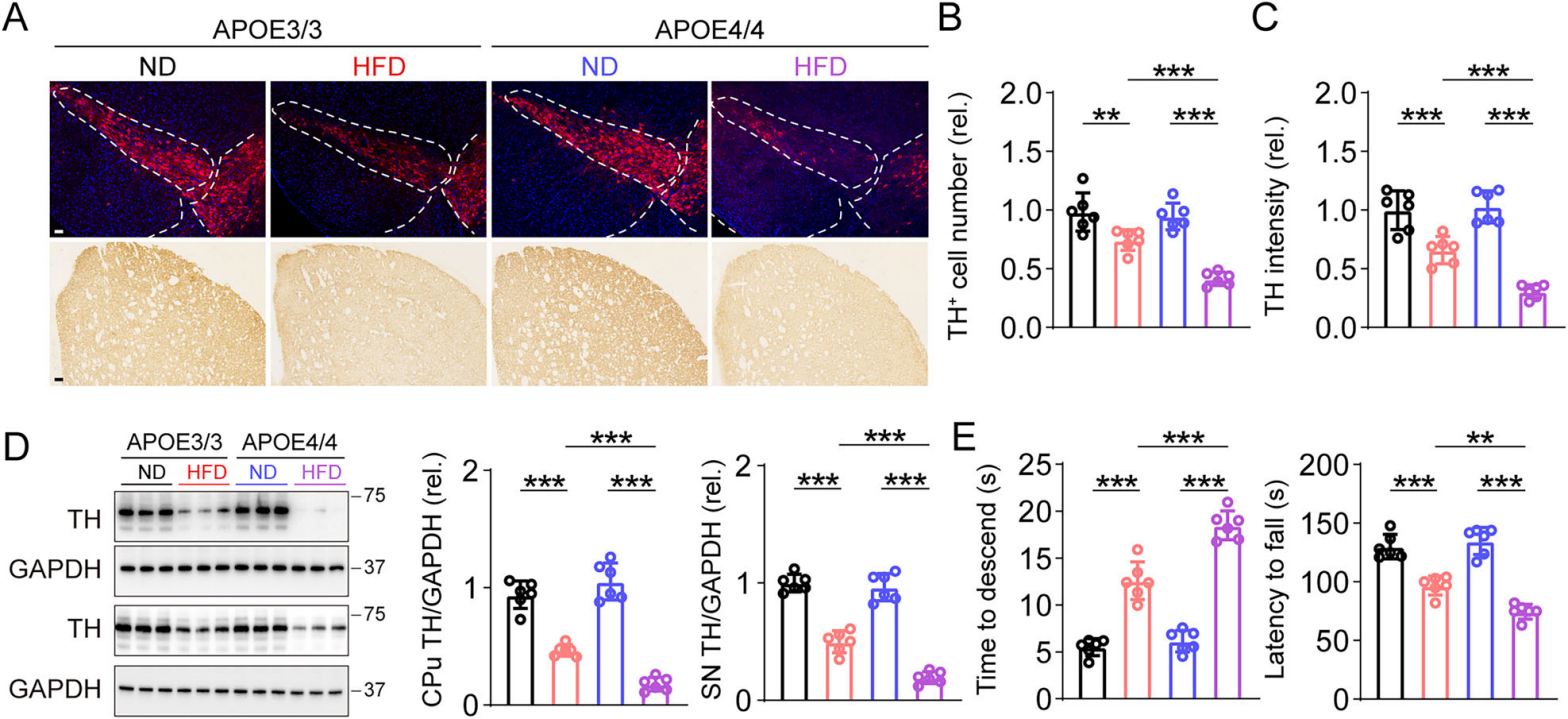
Nigrostriatal degeneration was more evident in APOE4/4 mice fed with high fat diet. **A** Representative TH IF and IHC staining in SNpc and CPu. Bar in upper panel, 40 μm, bar in lower panel, 50 μm. **B**, **C** Quantification of TH positive cell number and intensity in SNpc and CPu. *n* = 6, two-way ANOVA followed by Bonferroni’s post hoc tests. SN, Treatment: *F*(1, 20) = 69.73, *p* < 0.001, Genotype: *F*(1, 20) = 15.89, *p* < 0.001, Treatment × Genotype: *F*(1, 20) = 9.962, *p* = 0.005, CPu, Treatment: *F*(1, 20) = 106.3, *p* < 0.001, Genotype: *F*(1, 20) = 9.964, *p* = 0.005, Treatment × Genotype: *F*(1, 20) = 13.74, *p* = 0.001. **D** Representative blots and quantification of TH in CPu and SNpc. *n* = 6, two-way ANOVA followed by Bonferroni’s post hoc tests. CPu TH, Treatment: *F*(1, 20) = 246.2, *p* < 0.001, Genotype: *F*(1, 20) = 4.147, *p* = 0.055, Treatment × Genotype: *F*(1, 20) = 21.85, *p* < 0.001, SN TH, Treatment: *F*(1, 20) = 324.3, *p* < 0.001, Genotype: *F*(1, 20) = 23.13, *p* < 0.001, Treatment × Genotype: *F*(1, 20) = 14.58, *p* = 0.001. **E** Time to descend in pole test and latency to fall from an rotarod test. *n* = 6, two-way ANOVA followed by Bonferroni’s post hoc tests. Time to descend, Treatment: *F*(1, 20) = 265.2, *p* < 0.001, Genotype: *F*(1, 20) = 30.16, *p* < 0.001, Treatment × Genotype: *F*(1, 20) = 19.55, *p* < 0.001, Latency to fall, Treatment: *F*(1, 20) = 151.0, *p* < 0.001, Genotype: *F*(1, 20) = 5.171, *p* = 0.034, Treatment × Genotype: *F*(1, 20) = 12.55, *p* = 0.002.

Since APOE4-HFD mice exhibited tau hyperphosphorylation in nigral region. We examined nigrostriatal region integrity. TH positive neurons in SNpc and TH positive fiber densities in CPu were reduced in APOE4-HFD compared with APOE3-HFD (Fig. 3A–C). No differences of TH positive neurons or fibers were observed in APOE3-ND and APOE4-ND mice. Consistent with the immunostaining results, blots showed reduced TH protein levels in SNpc and CPu in APOE4-HFD mice versus APOE3-HFD (Fig. 3D). Pole test and rotarod test showed that motor impairments were more severe in APOE4-HFD mice compared with APOE3-HFD (Fig. 3E).

### Cholesterol induced tau hyperphosphorylation through GSK3β

**Fig. 4:**
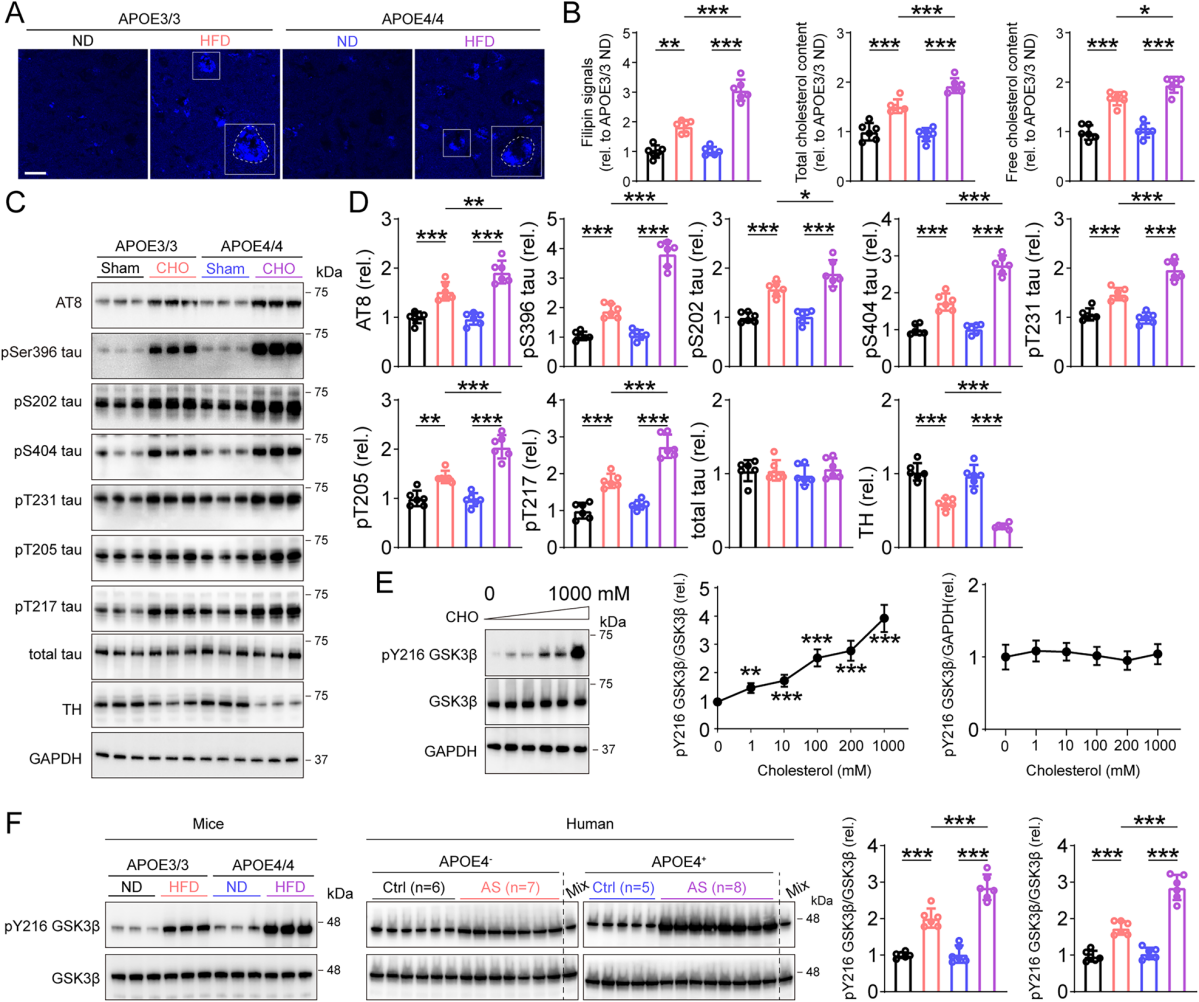
Cholesterol mediated tau hyperphosphorylation through GSK3β activation. **A** Filipin staining in SNpc. Bar, 30 μm. **B** Filipin intensity, serum total cholesterol and serum free cholesterol in AS mice model. *n* = 6, two-way ANOVA followed by Bonferroni’s post hoc tests. Filipin signals, Treatment: *F*(1, 20) = 218.9, *p* < 0.001, Genotype: *F*(1, 20) = 39.54, *p* < 0.001, Treatment × Genotype: *F*(1, 20) = 37.43, *p* < 0.001, Total cholesterol content, Treatment: *F*(1, 20) = 151.5, *p* < 0.001, Genotype: *F*(1, 20) = 8.453, *p* = 0.008, Treatment × Genotype: *F*(1, 20) = 15.10, *p* < 0.001, Free cholesterol content, Treatment: *F*(1, 20) = 180.9, *p* < 0.001, Genotype: *F*(1, 20) = 7.511, *p* = 0.012, Treatment × Genotype: *F*(1, 20) = 3.942, *p* = 0.061. **C**, **D** Representative blots and quantification of phosphorylated tau at multiple sites in primary cultured neurons treated with cholesterol. *n* = 6, two-way ANOVA followed by Bonferroni’s *post ho*c tests. AT8, Treatment: *F*(1, 20) = 112.6, *p* < 0.001, Genotype: *F*(1, 20) = 6.912, *p* = 0.016, Treatment × Genotype: *F*(1, 20) = 8.925, *p* = 0.007, pSer396 tau, Treatment: *F*(1, 20) = 269.3, *p* < 0.001, Genotype: *F*(1, 20) = 80.02, *p* < 0.001, Treatment × Genotype: *F*(1, 20) = 74.42, *p* < 0.001, pS202 tau, Treatment: *F*(1, 20) = 103.3, *p* < 0.001, Genotype: *F*(1, 20) = 5.672, *p* = 0.027, Treatment × Genotype: *F*(1, 20) = 4.229, *p* = 0.053, pS404 tau, Treatment: *F*(1, 20) = 240.3, *p* < 0.001, Genotype: *F*(1, 20) = 41.68, *p* < 0.001, Treatment × Genotype: *F*(1, 20) = 37.75, *p* < 0.001, pT231 tau, Treatment: *F*(1, 20) = 138.5, *p* < 0.001, Genotype: *F*(1, 20) = 12.74, *p* = 0.001, Treatment × Genotype: *F*(1, 20) = 23.27, *p* < 0.001, pT205 tau, Treatment: *F*(1, 20) = 118.4, *p* < 0.001, Genotype: *F*(1, 20) = 17.79, *p* < 0.001, Treatment × Genotype: *F*(1, 20) = 21.18, *p* < 0.001, pT217 tau, Treatment: *F*(1, 20) = 174.3, *p* < 0.001, Genotype: *F*(1, 20) = 36.46, *p* < 0.001, Treatment × Genotype: *F*(1, 20) = 18.47, *p* < 0.001, total tau, Treatment: *F*(1, 20) = 0.662, *p* = 0.4253, Genotype: *F*(1, 20) = 0.079, *p* = 0.781, Treatment × Genotype: *F*(1, 20) = 0.569, *p* = 0.459, TH, Treatment: *F*(1, 20) = 179.8, *p* < 0.001, Genotype: *F*(1, 20) = 18,35, *p* < 0.001, Treatment × Genotype: *F*(1, 20) = 9.291, *p* = 0.006. **E** Representative blots of phosphorylated GSK3β at Y216 site and total GSK3β in primary cultured neurons treated with cholesterol. One-way ANOVA followed by Bonferroni’s post hoc tests. pY216GSK3β, *F*(5, 30) = 76.73, *p* < 0.001. GSK3β, *F*(5, 30) = 0.7353, *p* = 0.6028. **F** Representative blots of pY216 GSK3β and total GSK3β in AS mice and postmortem AS patients brain SNpc. Mice, *n* = 6, two-way ANOVA followed by Bonferroni’s post hoc tests.Treatment: *F*(1, 20) = 192.4, *p* < 0.001, Genotype: *F*(1, 20) = 18.02, *p* < 0.001, Treatment × Genotype: *F*(1, 20) = 15.62, *p* < 0.001. Human, *n* = 5–8, two-way ANOVA followed by Bonferroni’s post hoc tests. Treatment: *F*(1, 22) = 199.4, *p* < 0.001, Genotype: *F*(1, 20) = 41.67, *p* < 0.001, Treatment × Genotype: *F*(1, 22) = 31.97, *p* < 0.001. *p < 0.05, **p < 0.01, and ***p < 0.001.

Since cholesterol regulation was affected by APOE genotype. We next measured cholesterol contents in nigral region and serum, respectively. Staining intensity of cholesterol by filipin was comparable in APOE3-ND and APOE4-ND mice, but significantly increased in APOE4-HFD mice versus APOE3-HFD (Fig. 4A, B). Moreover, increase in free and total cholesterol contents were observed in serum of APOE4-HFD mice compared with APOE3-HFD (Fig. 4B). These findings again suggest a important role of APOE genotype in regulating cholesterol contents.

Based on the possible effects of cholesterol on tau hyperphosphorylation in APOE4 mice treated with HFD. We treated primary cultured neurons from APOE4/4 and APOE3/3 mice with cholesterol, respectively. Significantly increases in different p-tau epitopes (AT8, pS396 tau, pS202 tau, pS404 tau, pT231 tau, pT205 tau and pT217 tau) were seen in APOE4-CHO neurons versus APOE3-CHO (Fig. 4C, D).

Given that GSK3β activation was associated with tau hyperphosphorylation, we subsequently treated primary cultured neurons with cholesterol at 0-1000 mM gradient. A significantly increase levels of active form of GSK3β, namely phosphorylation at the Tyr 216 site, were seen as the concentration of cholesterol increasing (Fig. 4E). Further tests confirmed that the pY216 GSK3β were increased in APOE4-HFD mice and APOE4^+^ AS patients brains versus APOE3-HFD mice and APOE4^-^ AS patients respectively (Fig. 4F).

### Reducing cholesterol alleviated nigrostriatal pathology and motor performances in APOE4 mice treated with HFD

**Fig. 5:**
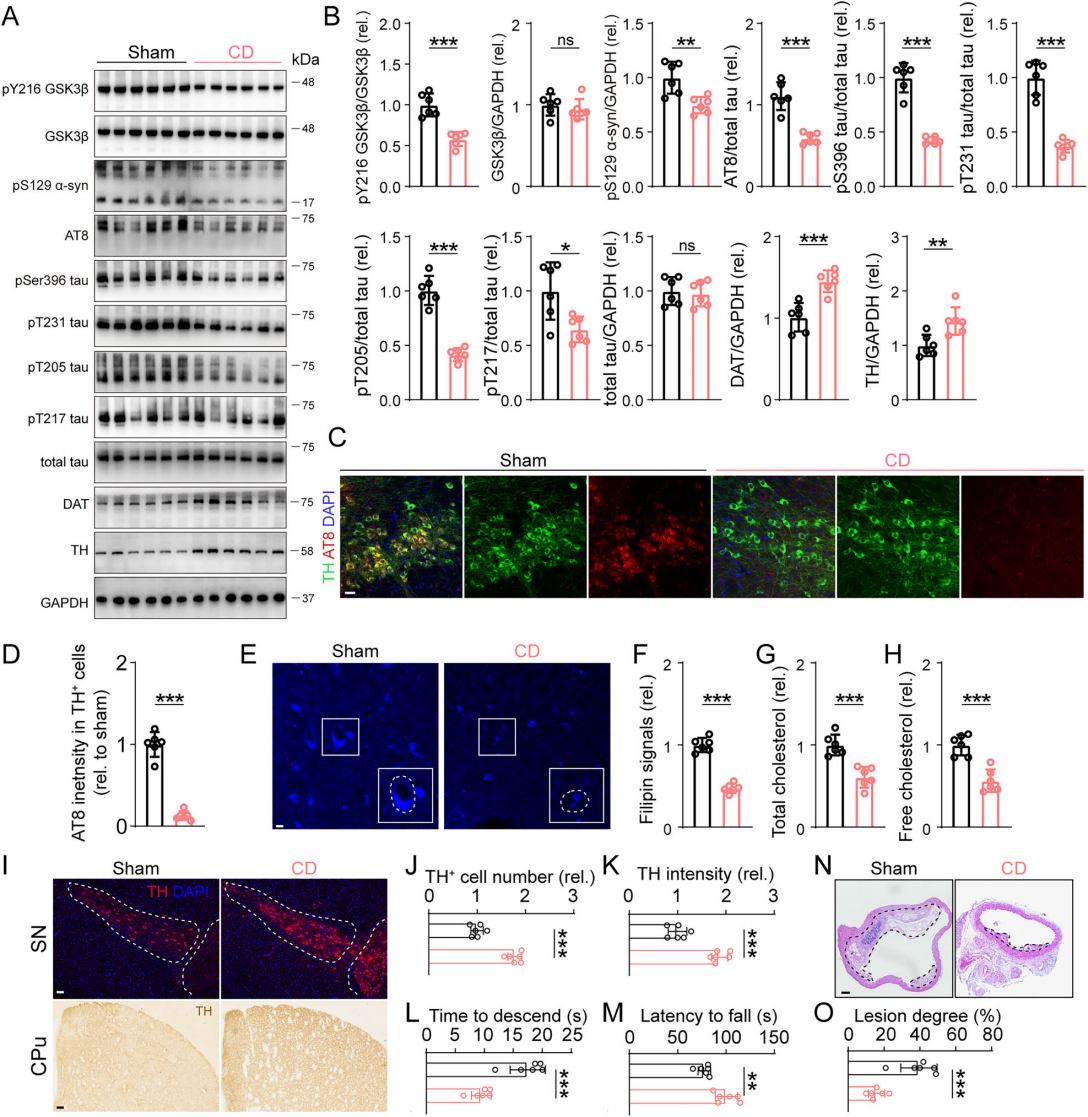
HP-β-CD alleviated tau pathology and motor impairments in AS mice. **A**, **B** Representative blots and quantification of phosphorylated tau at multiple sites, pY216 GSK3β and pSer129 α-syn in SNpc of AS mice. *n* = 6, unpaired student’s *t* test, pY216 GSK3β: *t*(10) = 6.281, *p* < 0.001, GSK3β: *t*(10) = 0.716, *p* = 0.49, pSer129 α-syn: *t*(10) = 3.734, *p* = 0.004, AT8: *t*(10) = 6.913, *p* < 0.001, pSer396 tau: *t*(10) = 10.28, *p* < 0.001, pThr231 tau: *t*(10) = 9.157, *p* < 0.001, pThr205 tau: *t*(10) = 9.893, *p* < 0.001, pThr217 tau: *t*(10) = 2.994, *p* = 0.014, total tau: *t*(10) = 0.3543, *p* = 0.7305, DAT: *t*(10) = 4.908, *p* < 0.001, TH: *t*(10) = 3.434, *p* = 0.006. **C** Double IF staining of TH and AT8 in SNpc. Bar, 20 μm. **D** Relative AT8 intensity in TH positive cells. *n* = 6, unpaired student’s *t*test, *t*(10) = 13.26, *p* < 0.001. **E** Filipin staining in SNpc of AS mice. Bar, 5 μm. **F** Filipin intensity analysis. *n* = 6, unpaired student’s *t*test, *t*(10) = 12.66, *p* < 0.001. **G**, **H** Serum total cholesterol and serum free cholesterol in AS mice treated with HP-β-CD. *n* = 6, unpaired student’s *t* test, total cholesterol, *t*(10) = 5.372, *P* < 0.001, free cholesterol, *t*(10) = 5.742, *p* < 0.001. **I–K** Representative TH staining and quantification of TH positive cell number and intensity in SNpc and CPu, respectively. Bar in upper panel, 50 μm, bar in lower panel, 40 μm. *n* = 6, unpaired student’s *t* test, SNpc, *t*(10) = 9.824, *p* < 0.001, CPu, *t*(10) = 8.315, *p* < 0.001. **L**, **M** Time to descend in pole test and latency to fall from an rotarod test. *n* = 6, unpaired student’s *t* test, time to descend, *t*(10) = 5.597, *p* < 0.001, latency to fall, *t*(10) = 4.242, *p* = 0.002. **N**, **O** Representative HE staining of mice aorta. Black dashed line showed that area of plaque. Bar, 100 μm. *n* = 6, unpaired student’s *t* test, *t*(10) = 5.188, *p* < 0.001.

Since cholesterol accumulation was able to trigger tau related pathology and motor impairments, we therefore reasoned that aiding cholesterol transport might exert protective effects in APOE4-HFD mice. To test this hypothesis, we employed 2-hydroxypropyl-β-cyclodextrin (2hβCD), which facilitate reducing intracellular cholesterol contents. Eight weeks of 2hβCD subcutaneous injections reduced the p-tau levels in APOE4-HFD mice accompanied by GSK3β inactivation (Fig. 5A, B). And TH and DAT protein levels were rescued (Fig. 5A, B). Correlated with these results, IF staining showed a reduction of AT8 intensity in 2hβCD treated APOE4-HFD mice (Fig. 5C, D). The filipin intensity in nigral region, serum free cholesterol and total cholesterol were reduced in APOE4-HFD mice after 2hβCD injection (Fig. 5E–H). Nigrostriatal pathologies were alleviated in 2hβCD treatment mice by TH immunostaining in SNpc and CPu (Fig. 5I, J, K). Motor impairment in pole test and rotorad test were diminished by 2hβCD injection (Fig. 5L, M). Lesion degrees of aortas were alleviated by 2hβCD (Fig. 5N, O).

## Discussion

Our observations demonstrated the role of cholesterol on tau hyperphosphorylation in APOE4/4 AS mice and APOE4 carried AS patients. In APOE4 carried AS patients, nigrostriatal degeneration and p-tau accumulation were observed. Further dissection of mechanisms revealed that nigral cholesterol dysregulation in APOE4 AS mice model. The accumulation of cholesterol caused tau hyperphosphorylation through kinase GSK3β activation. The DA neuronal p-tau aggregation triggered nigrostriatal degeneration, leading to motor impairment in AS mice.

AS had been shown to potentiate parkinsonism in old age [3]. And a shared molecular mechanism was recognized in AS and PD [15]. In the current study, we found DA neuron loss, glial activation and α-syn hyperphosphorylation were found in postmortem AS patient nigral regions. In particular, APOE4 potentiated the severity of nigrostriatal pathology compared with non-APOE4 carried AS patients. These observations paralleled those findings in which APOE4 mediated mitophagy impairments promoted parkinsonism in human brains [26, 27]. Postmortem human brain studies showed tau hyperphosphorylation precedes Lewy body formation in SNpc [28]. And genome-wide association studies showed *MAPT* (encodes tau protein) was associated with parkinsonism [29]. Thus, we tested the nigral p-tau levels in AS patients brains. To our surprise, tau at multiple sites including AT8 (Ser202 and Thr205) and Ser396 were hyperphosphorylated in AS patients relative to healthy controls. Critically, p-tau levels were significantly increased in APOE4 carriers relative to non-APOE4 carriers. Our finding of interactions of APOE4 and tau hyperphosphorylation shed insights into the pathophysiological mechanisms between AS and PD.

APOE3/3 and APOE4/4 knocking in mice were employed to validate the existing of nigral tau phosphorylation in AS pathology. Firstly, we treated APOE3/3 and APOE4/4 mice with HFD to model AS pathology. After 8 weeks of HFD feeding, APOE3 and APOE4 mice displayed AS pathology in aortic arteries compared with normal diet controls. Note that APOE4-HFD mice had a relative narrower vascular lumen compared with APOE3-HFD mice. IB results showed that tau hyperphosphorylation were evident in APOE3-HFD and APOE4-HFD mice. Moreover, IF images showed that the p-tau was selectively accumulated in DA neurons. We speculated the accumulated p-tau might trigger DA neurodegeneration in AS mice model. To validate our hypothesis, we assessed the TH protein expression in nigrostriatal system. The DA neurodegeneration and motor impairment were observed in HFD treated mice relative to ND controls. Note that the nigrostriatal pathology and motor function defects were more evident in APOE4-HFD relative to APOE3-HFD mice. These findings collectively suggested that AS mice exhibited nigrostriatal degeneration and motor function impairment.

Cholesterol dysregulation had been reported to be related to APOE4 genotype [30–32]. And we found cholesterol contents were increased in AS mice compared with controls. We hypothesized that tau might be hyperphosphorylated in the presence of cholesterol. We treated primary cultured neurons with cholesterol and found cholesterol triggered tau phosphorylation in APOE3 and APOE4 primary cultured midbrain neurons. As GSK3β was a major kinase for tau phosphorylation [33–35], we treated primary neuron with different concentrations of cholesterol and found that cholesterol activated the GSK3β directly. The increase of activated GSK3β level were observed in APOE4-HFD mice and APOE4^+^ AS human brains relative to APOE3-HFD mice and APOE4^-^ AS brains, respectively. These results showed that cholesterol might induce tau hyperphosphorylation through GSK3β activation.

Cholesterol accumulation were observed in AS mice brain. We therefore reasoned that aiding cholesterol may alleviate AS and PD pathology. To test this hypothesis, CD were used to reduce neuronal cholesterol accumulation. We found pharmacological interventions alleviated AS pathology, nigral tau hyperphosphorylation, and motor function impairments in APOE4-HFD AS mice.

Indeed, our study had certain limitations and weakness based on the current results. First, other kinases and phosphatase that regulated tau phosphorylation state should be figure out through high throughout analysis. Nevertheless, we found a major kinase GSK3β which was activated by cholesterol directly. Second, we did not eliminate gender bias in this study. In future, more sophisticated researches employing proteomics and gender specific analysis should be used to fully describe the potential links between AS and PD.

In sum, base on our available evidence, we demonstrated that AS pathology was a risk factor for tau related PD pathology in APOE4 carriers. The neuronal cholesterol accumulation was able to trigger tau hyperphosphorylation through GSK3β activation in APOE4 carriers. Pharmacological intervention focused on facilitate cholesterol transport may alleviate the AS and PD pathology progression. These evidence shed more insights into the mechanism that AS coupled PD and provided potential therapeutic targets for treating AS and PD simultaneously.

## Materials and methods

### Postmortem human brain and coronary artery samples

A total of 26 postmortem human samples including brain SN and coronary arteries were acquired from school of forensic medicine of Guizhou Medical University. Samples were stored in liquid nitrogen or in 4% paraformaldehyde for further experiments. Clinical information were listed in supplementary file Table S1. All procedures were conducted according to the Ethics Committee of Guizhou Medical University (Approval number: 2023-91).

### Animals

APOE ε3/ε3 (APOE3) and APOE ε4/ε4 (APOE4) mice were purchased from Jackson Laboratory. The AS mice model was established using 10 months old APOE3 and APOE4 mice feeding with high fat diet (HFD) (hereafter APOE3 + HFD and APO4 + HFD, respectively) for 4 months. All experiments were approved by Zunyi Medical University Animal Care and Use Committee.

### Hematoxylin-eosin (HE) and immunohistochemistry (IHC) staining

The fixed human coronary arteris, brain SN tissues, mice aortic arteris and mice brains were dehydrated and embeded in wax. Three-µm sections of tissues were conducted using a microtome (RM22535, Leica, Germany). Briefly for HE staining, the sections were staining with hematoxylin for 1 min and washed with PBS. And then the sections were staining with eosin for 30 sec followed by dehydration. For IHC staining, the sections were rinsed in 0.01 M sodium citrate (Cat#C1010, Solarbio life sciences, China) for antigen retrieval followed by blocking in 3% hydrogen peroxide (Cat#7722-84-1, Maclin Inc, China) for 10 min to diminish the endogenous peroxidase. Then sections were incubated with primary antibodies (summarized in supplementary file Table S2) overnight at 4 °C. Sections were developed with a streptavidin-HRP DAB kit (Cat#CW2069, CW bio, China). Images were acquired using a wide field microscope (Axio Observer 7, Zeiss, Germany).

### Primary midbrain neuron culture

Postnatal APOE3 and APOE4 mice (1 ~ 2 days) midbrain tissues were acquired and then digested. The Neurobasal-A (10888022, Gibico, Invitrogen) and B27 (175044-044, Gibico, Invitrogen) were used to culture the neurons. Different concentrations of cholesterol (Cat#C4951, Sigma-Aldrich, USA) was added into the medium for 1 h. Neurons were collected and kept in –80 °C for subsequent analysis.

### Immunoblotting (IB)

Total proteins from cultured cells and brain tissues were homogenized using RIPA lysis buffer (Cat#P0013 and P0013B, Beyotime Biotechnology, China) supplemented with protease and phosphatase inhibitors (Cat#P1050, Beyotime Biotechnology; Cat#AC0220 and AC0224, CINOTOHI). The homogenized lysates were incubated on ice for 30 min, sonicated 3 times and centrifuged at 12,000 rpm (4 °C) for 20 min. The supernatant was collected for downstream analysis. Protein concentrations were determined using a BCA protein assay kit (Cat#P0010S, Beyotime Biotechnology). The protein samples were mixed with 5 × SDS-PAGE loading buffer (Leagene, PE0025-1), heated at 100 °C in a metal bath for 7 min, and briefly cooled. Proteins were resolved on 10 ~ 12% SDS-polyacrylamide gels and transferred to PVDF membranes (Cat#ISEQ00010, Millipore) using standard wet transfer conditions. Membranes were blocked with 5% non-fat milk in TBST for 1 h at room temperature. Primary antibodies (summarized in supplementary file Table S2) were diluted in blocking buffer and incubated overnight at 4 °C. After washing with TBST, membranes were probed with HRP-conjugated secondary antibodies for 1 hr at room temperature. Protein bands were visualized using a SuperEnhanced ECL reagent kit (GBCBIO, G3308) and imaged with a chemiluminescence detection system. Band intensities were quantified using ImageJ software.

### Immunofluorescence (IF) staining

The brain tissues were fixed in 4% paraformaldehyde for 48 h and then were sectioned (40 µm in thickness) using a cryostat microtome (CM1950, Leica, Germany). The free floating sections were blocked in 5% bovine serum albumin for 40 min. Sections were incubated in primary antibodies including mouse anti-pSer202 & Thr205 tau (AT8, MN1020, 1:800 dilution, Thermofisher Scientific, USA), rabbit anti-TH (EP1532Y, 1:1000 dilution, Abcam, USA) and pSer396tau (EPR2371, 1:800 dilution, Abcam, USA) after washing in 0.1 M PBS. After incubated with appropriate secondary antibodies (A21206/A10037, 1:500 dilution, Thermofisher Scientific, USA), sections were sealed with coverslips. Images were acquired using a confocal microscope (LSM880, Carl Zeiss, Germany). One average was plotted for the five images taken across the sections for one animal. The number of the animals in each group were indicated in the figure legends.

### Filipin staining

Briefly, brain sections (40 µm in thickness) containing nigral region were acquired according to the protocols in IF staining above. Free flouting sections were stained using Filipin solution (0.25 mg/ml in PBS, Cat#F4767, Sigma-Aldrich, USA) for 15 min. After washed in 0.1 M PBS, sections were mounted on glasses. Images were acquired using a confocal microscope (LSM880, Carl Zeiss, Germany).

### Serum cholesterol analysis

The total and free cholesterol were analyzed using a cholesterol quantitation kit (ab65359, Abcam, USA) according to the manufacturer’s protocols. The cholesterol contents were calculated according to the standard curve.

### Behavioral test

For the pole test, an 1 cm diameter metal pole (length: 75 cm) wrapped with bandage gauze was fixed on the base upright. The mice were trained for two consecutive days before the true test. On the testing day, mice were placed 10 cm from the top of the pole facing head-up. The total time to reach the base of the pole were recorded.

For rotarod test, the mice were placed on a rotational cylinder. The speed of the rotarod cylinder was slowly increased from 0 to 40 r.p.m. within 5 min. The trial was ended if the mice fell off the cylinder and the duration on the cyliner was recorded. Mice were trained 3 days before true tests.

### Statistical analysis

Data were expressed mean ± sem. Depending on the data sets, statistical analysis were performed using Two-way ANOVA by Bonferroni’s post hoc analysis or unpaired Student’s *t* test using SPSS 22.0 (IBM, New York, USA) and/or GraphPad Prism 8 (GraphPad, USA). The *p* value was set at <0.05. Researchers were blinded to the experiments when conducting behavioral tests and pathological analysis. The statistical parameters including n, *T*, *P, r* and *F* were listed in the figure legends.

## Supplementary information

Supplementary file

## Data Availability

The datasets during and/or analyzed during the current study are available from the corresponding author on reasonable request.
