# Supplementary material for: APOE4 promotes nigral tau hyperphosphorylation through cholesterol in atherosclerosis
Source: Cell Death Discov. 2025 Oct 21;11:478. doi: 10.1038/s41420-025-02778-1 (PMC12540723; doi:10.1038/s41420-025-02778-1)

**Table S1. Information of human samples**

| Group | Gender | Age (year) | Comorbidity | COD | PMI (hrs) |
| --- | --- | --- | --- | --- | --- |
| APOE4- control | M | 45 | NA | Hemorrhagic shock | 33 |
| APOE4- control | M | 78 | NA | Multiple organ dysfunction syndrome | 29 |
| APOE4- control | F | 39 | NA | hemorrhagic shock | 42 |
| APOE4- control | M | 69 | Hypertension | Fall | 31 |
| APOE4- control | F | 49 | NA | Pulmonary embolism | 50 |
| APOE4- control | F | 82 | Diabetes mellitus;  Hypertension | Multiple organ dysfunction syndrome | 26 |
| APOE4- AS | M | 62 | Coronary atherosclerosis | Acute myocardial infarction | 19 |
| APOE4- AS | F | 69 | Coronary atherosclerosis;  Hypertension | Acute myocardial infarction | 18 |
| APOE4- AS | M | 73 | Coronary atherosclerosis;  Hypertension | Acute myocardial infarction | 10 |
| APOE4- AS | M | 62 | Coronary atherosclerosis, hyperlipidemia | Acute myocardial infarction | 18 |
| APOE4- AS | F | 80 | Coronary atherosclerosis | Acute myocardial infarction | 21 |
| APOE4- AS | F | 73 | Coronary atherosclerosis, hyperlipidemia | Acute myocardial infarction | 24 |
| APOE4+ control | F | 63 | NA | Hemorrhagic shock | 24 |
| APOE4+ control | F | 46 | NA | Fall | 19 |
| APOE4+ control | F | 51 | NA | hemorrhagic shock | 25 |
| APOE4+ control | F | 82 | Diabetes mellitus; hyperlipidemia | Electric shock | 26 |
| APOE4+ control | M | 79 | Hypertension, | Hemorrhagic shock | 39 |
| APOE4+ AS | F | 73 | Coronary atherosclerosis;  Pneumonia | Acute myocardial infarction | 42 |
| APOE4+ AS | F | 82 | Coronary atherosclerosis;  Diabetes mellitus | Acute myocardial infarction | 27 |
| APOE4+ AS | F | 76 | Coronary atherosclerosis;  Diabetes mellitus | Acute myocardial infarction | 31 |
| APOE4+ AS | F | 73 | Coronary atherosclerosis | Acute myocardial infarction | 50 |
| APOE4+ AS | F | 69 | Coronary atherosclerosis | Acute myocardial infarction | 24 |
| APOE4+ AS | M | 58 | Coronary atherosclerosis;  Diabetes mellitus;  Hypertension | Acute myocardial infarction | 31 |
| APOE4+ AS | F | 57 | Coronary atherosclerosis | Acute myocardial infarction | 21 |
| APOE4+ AS | F | 69 | Coronary atherosclerosis;  Hypertension | Acute myocardial infarction | 24 |

**Abbreviations:** Gender: F, female; M, male. NA, non-applicable. COD, cause of death. PMI, postmortem interval.

**Table S2. Antibodies used in this study**

| Antibodies | Source | Identifier | Dilution |
| --- | --- | --- | --- |
| AT8 | ThermoFisher Scientific | Cat#MN1020 | WB, 1:1000; IHC, 1:200; IF, 1:800 |
| TH | Abcam | Cat#EP1532Y | WB, 1:1000; IHC, 1:1000; IF, 1:1000 |
| DAT | ThermoFisher Scientific | Cat#MA5-24796 | WB, 1:1000 |
| Phospho-α-synuclein Ser129 | ThermoFisher Scientific | Cat#PA1-4686 | WB, 1:1000; IHC, 1:200 |
| Phospho-Tau Ser396 | Abcam | Cat#EPR2371 | WB,1:1000; IF: 1:800 |
| Phospho-Tau Ser202 | ThermoFisher Scientific | Cat#PA5-121298 | WB,1:1000; |
| Phospho-Tau Thr205 | ThermoFisher Scientific | Cat#44-738G | WB,1:1000; |
| Phospho-Tau Thr214 | Affinity | Cat#AF3141 | WB,1:1000; |
| Phospho-Tau Thr217 | ThermoFisher Scientific | Cat#44-744 | WB,1:1000 |
| Phospho-Tau Thr231 | ThermoFisher Scientific | Cat#44-746G | WB,1:1000 |
| Phospho-Tau Thr181 | Cell Signaling Technology | Cat#12885 | WB,1:1000 |
| Phospho-Tau Ser404 | Cell Signaling Technology | Cat#20194 | WB,1:1000 |
| GSK3β | Abcam | Cat#ab32391 | WB,1:1000 |
| Phospho-GSK3β Tyr216 | Santa cruz biotechnology | Cat#05-413 | WB,1:1000 |
| Tau5 | Abcam | Cat#20194 | WB,1:1000 |
| GAPDH | Cell Signaling Technology | Cat#2118 | WB,1:1000 |
| GFAP | Cell Signaling Technology | Cat#3670 | IHC, 1:200 |
| Iba1 | Cell Signaling Technology | Cat#ab178846 | IHC, 1:300 |
| donkey anti-rabbit Alexa Fluor 488 | ThermoFisher Scientific | Cat#A21206 | IF, 1:500 |
| donkey anti-mouse Alexa Fluor 568 | ThermoFisher Scientific | Cat#A10037 | IF, 1:500 |

Uncropped blots


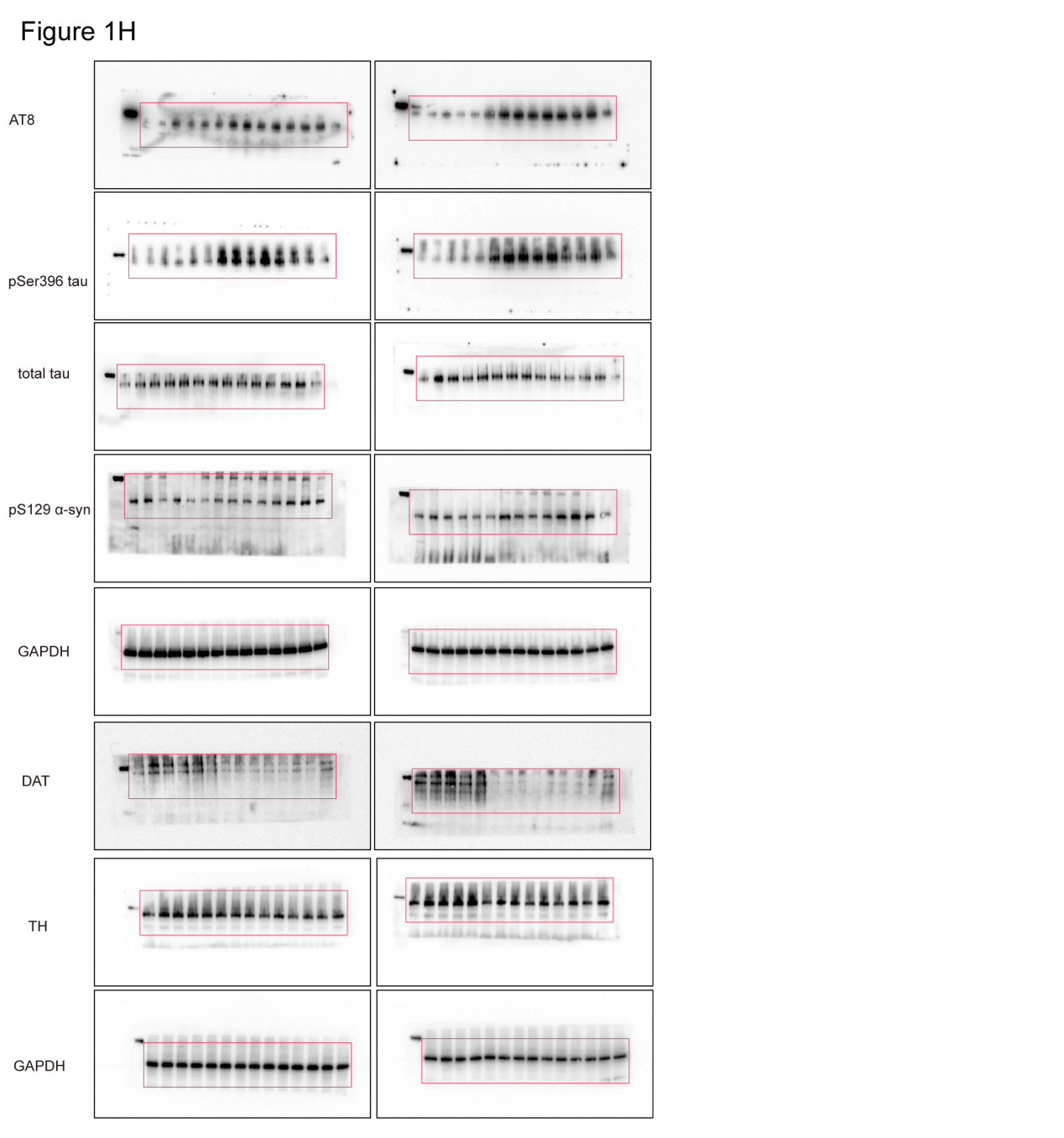


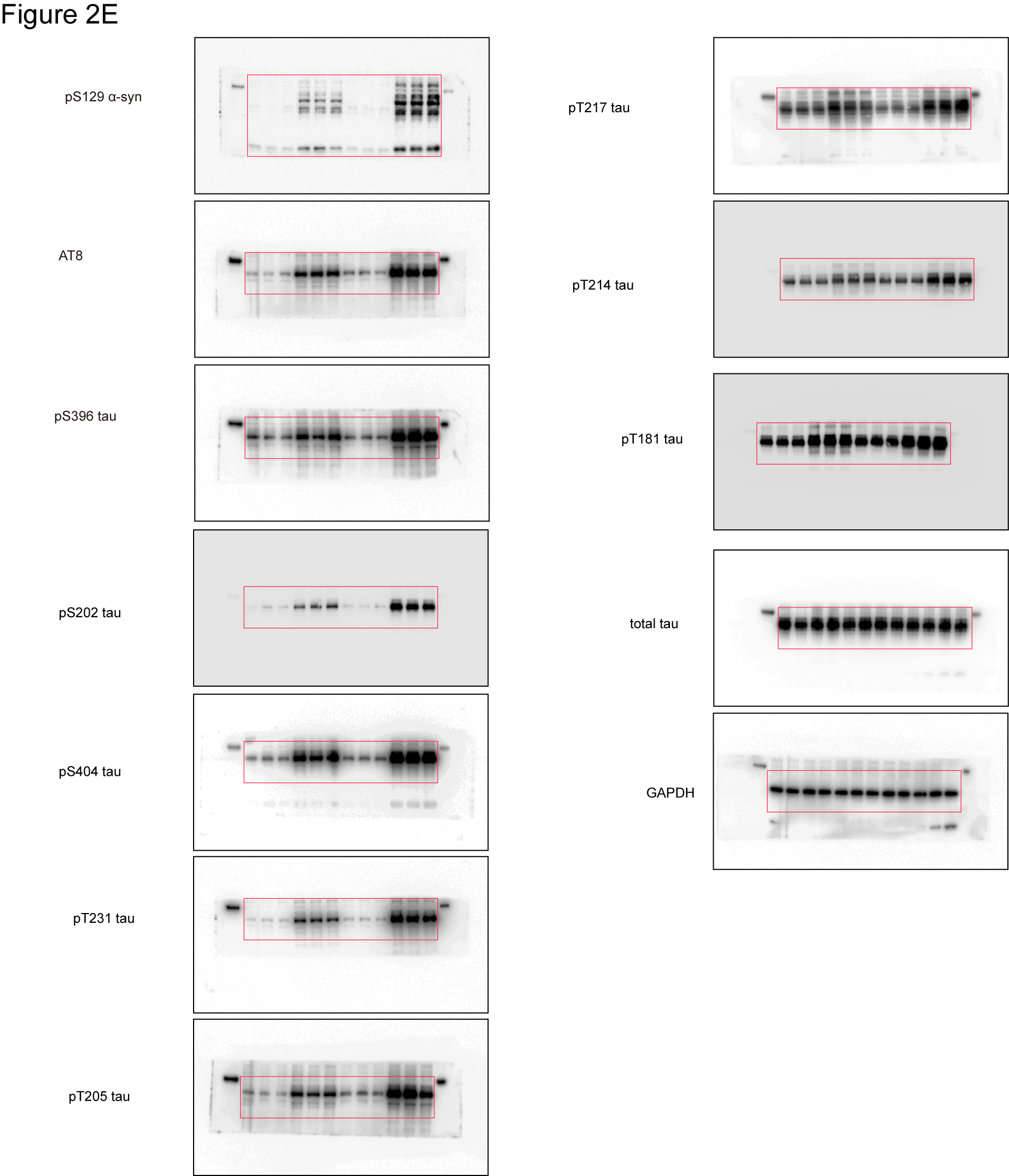


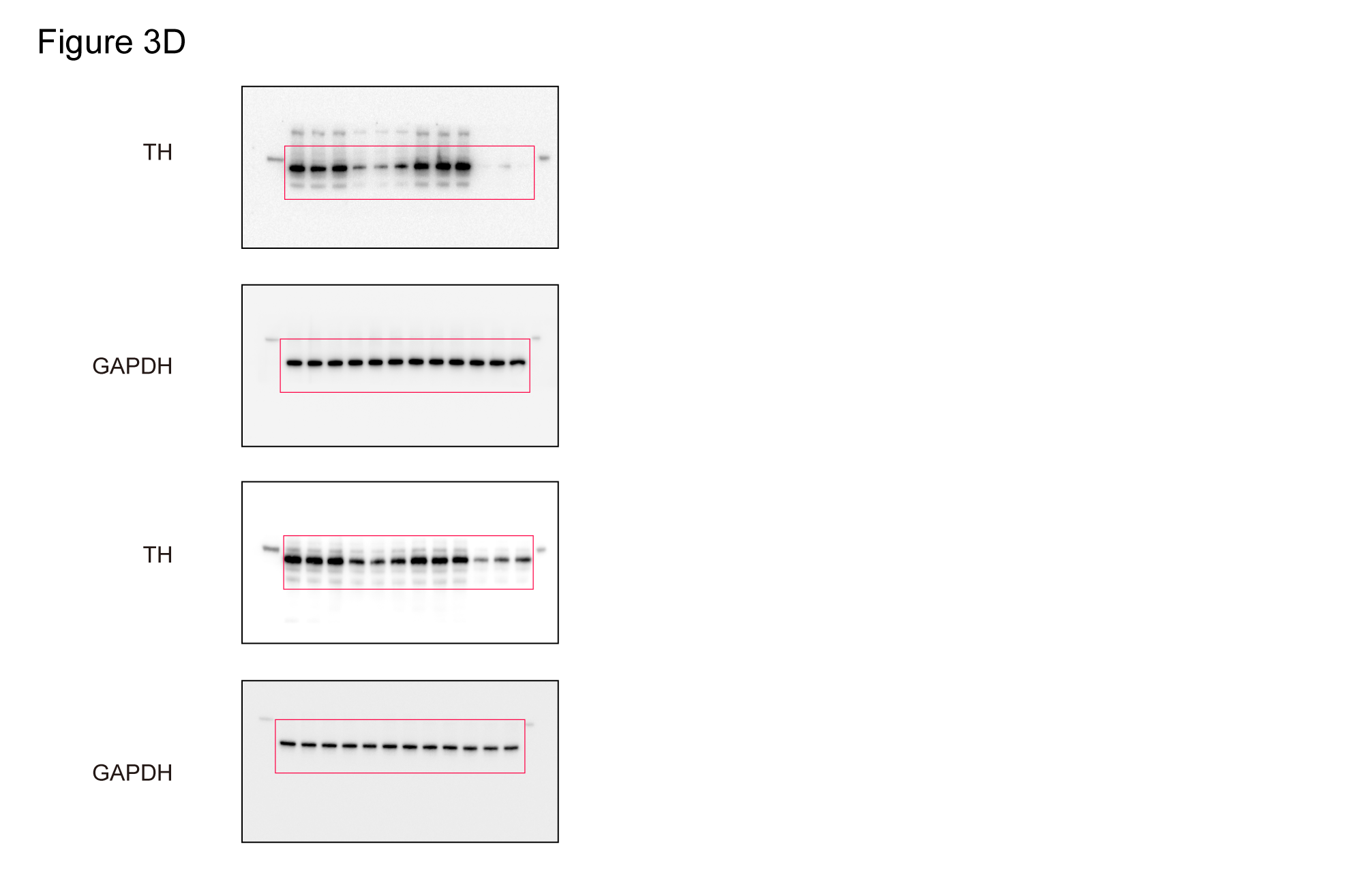


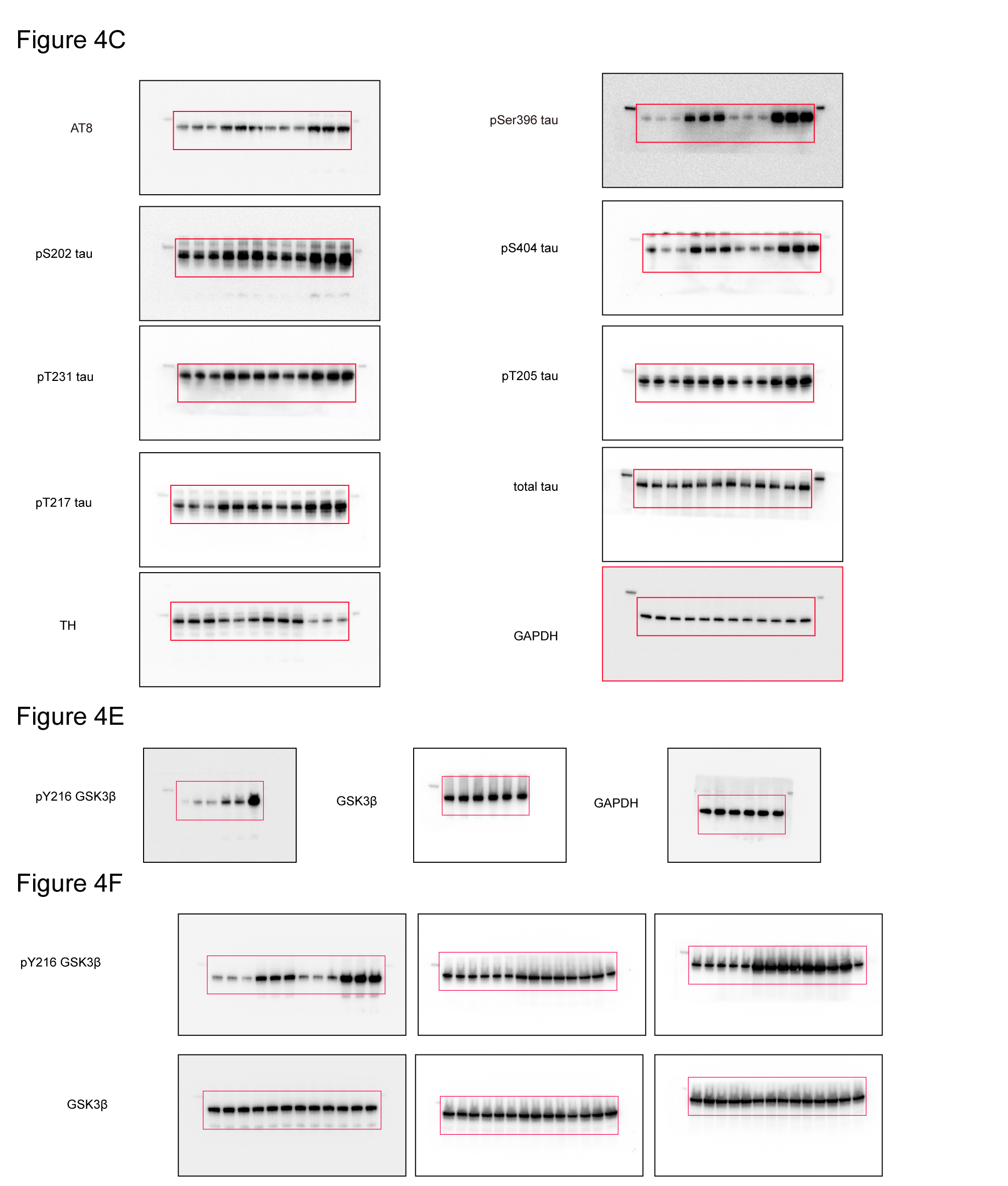


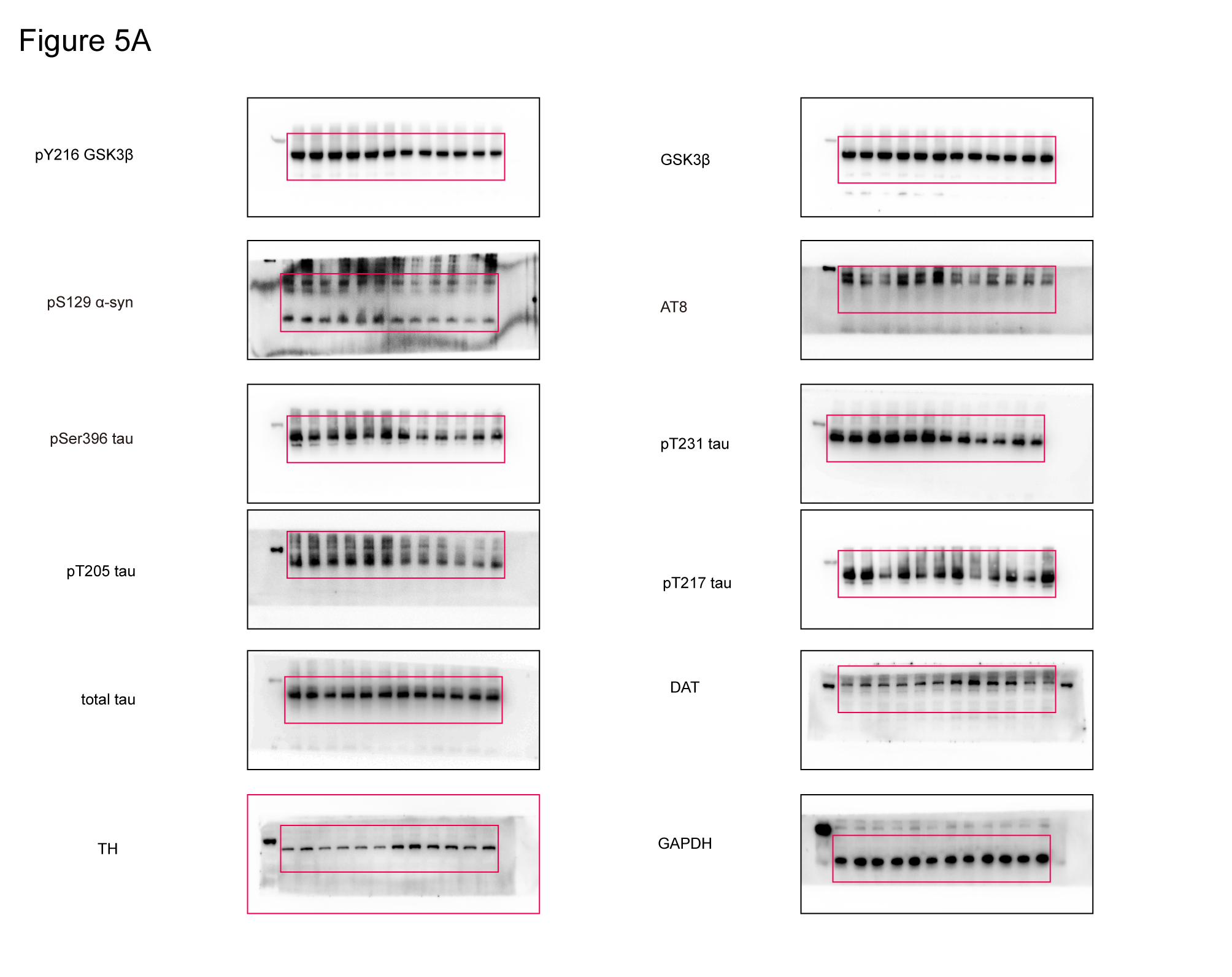

Supplement: Supplementary file 1 — Supplementary file [file 41420_2025_2778_MOESM1_ESM.docx]
